# Water availability and response of Tarbela Reservoir under the changing climate in the Upper Indus Basin, Pakistan

**DOI:** 10.1038/s41598-022-20159-x

**Published:** 2022-09-23

**Authors:** Firdos Khan

**Affiliations:** School of Natural Sciences (SNS), National University of Sciences and Technology (NUST), Islamabad, 44000 Pakistan

**Keywords:** Climate sciences, Hydrology

## Abstract

Agriculture is one of the major contributors to the Gross Domestic Product (GDP) of Pakistan which relies on the availability of water. Hydropower contributes approximately 35% to the national electricity gid of Pakistan. Indus River is the main river of the Indus River System (IRS) which provides water for agriculture, hydropower and other purposes. The outputs of the Conformal-Cubic Atmospheric Model (CCAM) are used to force the University of British Columbia Watershed Model (UBCWM) in the Upper Indus Basin (UIB), to investigate future water availability under the two IPCC emission scenarios (RCP4.5 and RCP8.5). Tarbela Reservoir which is the outlet of UIB is used as a measurement tool to assess water availability and response of the reservoir to climate change. The results show that maximum and minimum temperature are increasing in the future in comparison to the reference period. The largest increases in maximum temperature are projected for MAM (March–April–May) and JJA (June–July–August), with increases up to 2 °C in MAM and increases up to 6.4 °C in JJA under the RCP4.5 and RCP8.5, respectively, in the future. Minimum temperature has maximum increase (6.7 °C) in DJF (December–January–February) during 2071–2100 under RCP8.5. Precipitation shows a 5.1% decrease in DJF during 2011–2040 under RCP4.5. The statistics about water availability suggest that there is consistent increase in most of the months in the future, however, under the RCP4.5, there is decline in the river flow during 2071–2100 as compared to the 2041–2070. The findings of this study show that most of the time there will be more water available but in some months, there may be water scarcity under the RCP4.5, however, proper management and optimal utilization can reduce the water scarcity.

## Introduction

Climate change is a reality, and it can be seen from different events like heatwaves, extreme floods, drought etc., happened during the recent past decades^1–3^. In addition, it has adverse impacts on many sectors which are extremely important for our daily life. Water is one of them, without water the assumption of life is baseless. Due to global warming and climate change, higher population’s growth rate, increasing demand of water from industries, the demand of water increased and is expected to increase manifold in future^4^. Pakistan is in the list of top ten countries which are vulnerable to climate change and its worst impacts^5^. This makes it more important to investigate the future’s situation about water availability in Pakistan under the guidelines of the Intergovernmental Panel on Climate Change (IPCC).

The northern part of Pakistan is considered as a water tower for Pakistan since it comprises one of the world’s largest reserves of snow and ice outside the polar regions^6–8^. This part of the country provides water downstream for agriculture, hydropower generation, drinking and other purposes to the 5th most populous country of the world^9,10^. This region is more vulnerable to climate change as compared to the other parts of the country^11–14^. With the increase of 1 °C in mean summer temperature can increase the river flow by 16–17% in the Upper Indus Basin (UIB) and show the sensitivity of glaciers and snow melt in this region^15^. In recent studies researchers explored that in various parts of African countries and Pakistan, which are considered as climate change hotspots, anticipated changes in the heatwave frequency will affect the population^16–19^. Ullah et al.^20^ concluded that the exposure of the South Asian population to heatwaves is increasing in the future under the shared socioeconomic scenarios during the twenty-first century, however, the northern part of Pakistan is unaffected presently. Lutz et al.^21^ found out that climate change indicators under RCP4.5 and RCP8.5 show that the region may face strong adverse impacts of climate change for the hydropower production, occurrence of floods, human health, and agricultural production in the future.

Regarding precipitation, projected results show uncertainties over Pakistan^13,22^, however, some studies suggested that precipitation has mixed trend (increasing trend for some time period and decreasing trend for other time period) in the future over the northern part of Pakistan.

In Pakistan, the river flows have contribution from rainfall, glacier and snowmelt. The changing climate and changes in weather’s pattern can significantly change the river flows and consequently have impacts on agriculture, hydropower and other sectors in the country^23^. The contributions of glacier melting to river flow is difficult to assess because the former do not remain the same throughout the year, but studies show that in summer their contribution is almost 50–70%^24–26^.

There are two main reservoirs in Pakistan, Tarbela and Mangla constructed on River Indus and River Jhelum, respectively. Tarbela Dam is one of the world’s biggest dams with a rough storage capacity of 7 Million Acre Feet (MAF) and contributes 3400 megawatt (MW) electricity to the national electricity grid^27^. The focus of this study is Tarbela Reservoir, the main storage and control of the Indus irrigation system, which is one of the world’s largest irrigation networks^28^. Tarbela Dam is the outlet of UIB constructed on Indus River in Swabi and Haripur districts of Khyber Pakhtoonkhwa around 50 km North-West of Islamabad, the capital of Pakistan.

The Indus River provides 44% of the water for irrigation and other purposes annually^29,30^. It contributes 86% in summer season and 14% during winter season. Hydropower contributes 35% to the national electricity grid of Pakistan, where the Indus River itself contributes 79% at present and is expected to contribute 81% in the future^27^. This includes new hydropower projects with a capacity of at least 50 MW planned by Water and Power Development Authority (WAPDA)^27^. Due to the significant contribution of Indus River in terms of water and hydropower, naturally the question arises that what will be the situation about its water availability. This study aims to quantify the available water using different parameterizations of the Tarbela Reservoir in the UIB under a changing climate. In addition, the response of Tarbela reservoir is also assessed under the considered climate change scenarios.

**Figure 1:**
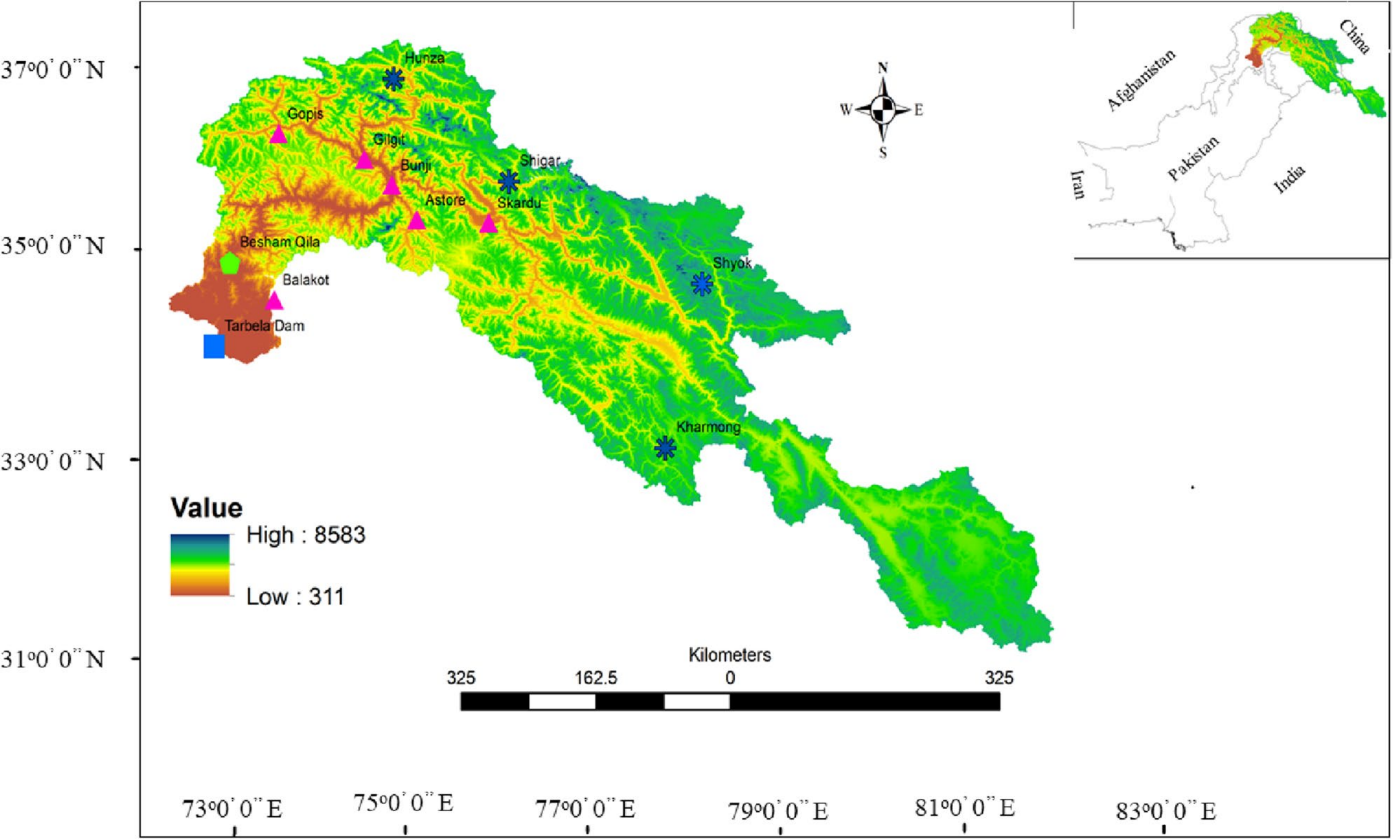
This figure show Pakistan and the Upper Indus Basin. The enlarged part shows the Upper Indus Basin where the meteorological stations are represented by pink tringles, subbasins by dark blue asterisk, Besham Qila by green pentagon and Tarbela Reservoir by blue square. This figure is created by using in ArcGIS version 10.6.1.

IPCC has developed guidelines in the form of different scenarios i.e., Representative Concentration Pathways (RCPs) with different emission levels. Choosing an RCP depends on the past and present climatology of the region of interest. Two climate change scenarios of the IPCC are being considered in this study i.e., RCP4.5 and RCP8.5. In these considered scenarios, one is medium level emission scenario (RCP4.5) and other is high level emission scenario (RCP8.5). In this study I investigate water availability by utilizing the simulated river flows, evapotranspiration, and forecasted reservoir outflow. The river flow is simulated by using University of British Columbia Watershed Model (UBCWM), reservoir outflow is forecasted by using Bayesian Dynamic Linear Model (BDLM) and evapotranspiration is calculated by using the method of Blaney and Criddle^31^. BDLM has the advantages over other time series models and can model a time series with no stationarity, structural breaks, sudden jumps and no clear pattern^32,33^. BDLM has useful applications in calculating the impacts of large-scale climate on droughts in Pakistan^34^, modelling and forecasting river flows in Brazil^35^ and exploring the sediment‐discharge rating curves in the New Yor, United States^36^. The University of British Columbia Watershed Model (UBCWM)^37–39^ has been used for river flow projections. Before simulating the river flow, the UBCWM is calibrated and validated for the Upper Indus Basin (UIB), with an approximate area of 164,000 km^2^. The details about the study area, its geographical location, and the locations of the hydro-meteorological stations used in this study are presented in Fig. 1.

The major aims of this study are: to investigate water availability in the Upper Indus Basin under the IPCC climate change scenarios; to make an assessment of the response of Tarbela Reservoir to the available water under the climate change scenarios. Furthermore, the findings of this study may help to address some of the Sustainable Development Goals (SDGs) of the United Nation Development Program (UNDP) related to water, famine and environment.

## Data sets

Different types of data set have been used, meteorological (maximum, minimum temperature and precipitation), hydrological (river flow), Digital Elevation Model (DEM), soil type and land use. The latter three types are used for the delineation of the basin, meteorological data is used to force UBCWM for river flow projections and the hydrological data is used for calibration, validation of the UBCWM. Brief information about the stations from which the climate data is used for deriving hydrological projections, is given in Table 1. The meteorological data for the duration of 1990–2004 is acquired from Pakistan Meteorological Department (PMD), river flow data for the time period of 1990–2004 is collected from WAPDA and Indus River System Authority (IRSA). The river flow data is divided into two independent sets, 1990–1999 and 2000–2004 and used for calibration and validation of the hydrologic model, respectively. The DEM data is collected from United States Geological Survey (USGS) while the land use and soil data is acquired from Food and Agriculture Organization (FAO).

**Table 1:** Brief information is presented about the stations from which the climate data is used for hydrological projections in the UIB.

S. no.	Station’s name	Latitude	Longitude	Elevation (m)	Missing values (%)
1	Astore	35.33	74.90	2168.0	1.5
2	Balakot	34.55	72.35	995.40	0.85
3	Bunji	35.67	74.63	1372.0	5.3
4	Gilgit	35.92	74.33	1460.0	3.8
5	Gopis	36.17	73.4	2156.0	4
6	Skardu	35.30	75.68	2317.0	4

The total number of observations is 5479 for the duration of 1990–2004 including missing values.

## Methodology

**Figure 2:**
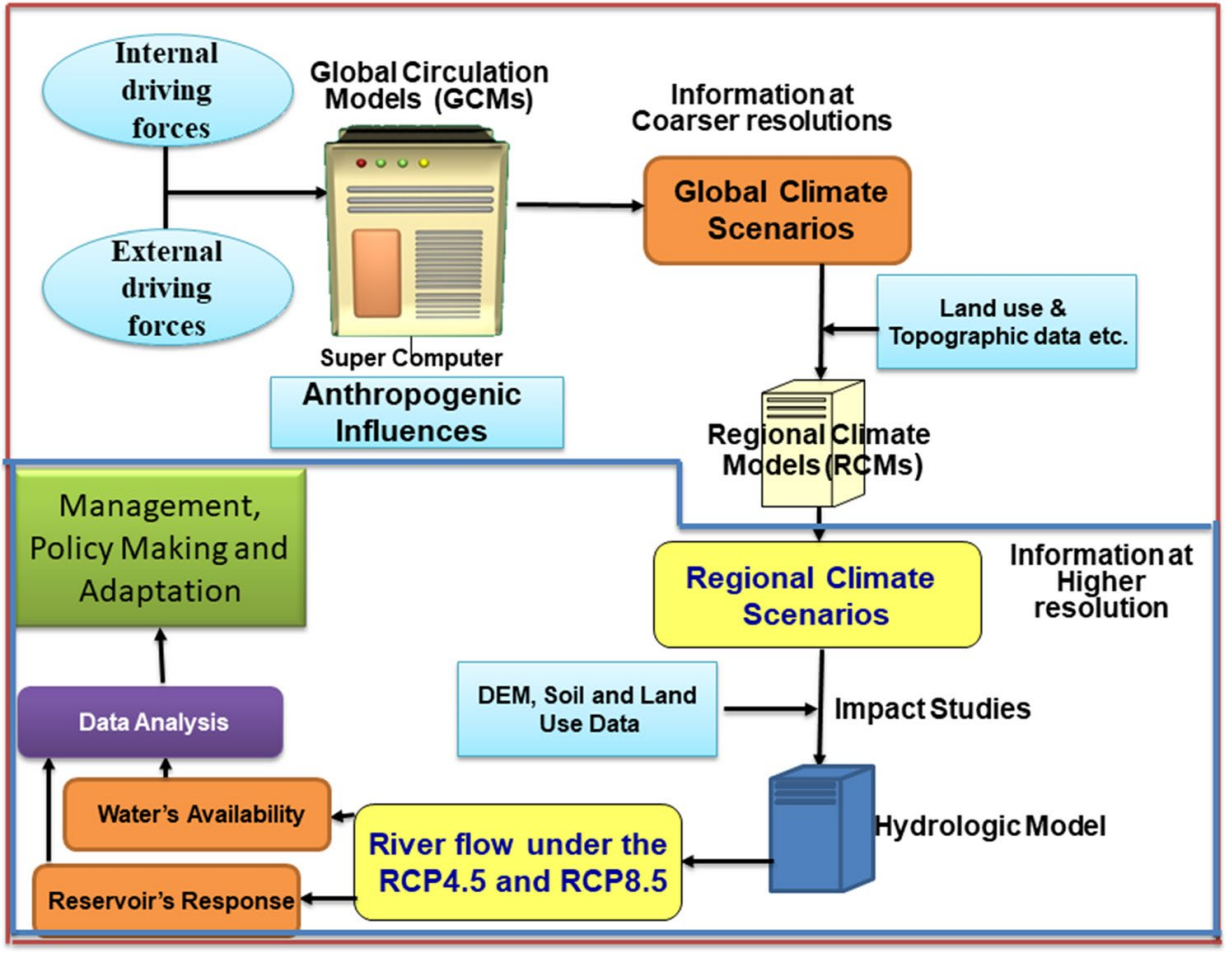
Schematic presentation of the methodology of the study to reach the objectives. The activities within blue line were performed for this study.

Major parts of the methodology are described in detail in the subsequent sections. The detailed and step-by-step methodology is presented in Fig. 2.

### Statistical bias correction

Climate models developed for the numerical simulation of physical processes in the land, ocean, and atmosphere are useful tools for climate predictions. However, these climate models involve assumptions and parameterizations for the simulation of complex process, which lead to structural and random errors called biases^40–43^. Several bias correction and evaluation approaches have been developed and applied in different studies to improve the quality and accuracy of RCMs’ outputs^44–47^. The results of^22^ suggested against the use of direct RCM and GCM outputs for the impact assessment studies over Pakistan, due to the biases in the outputs of these models. For further details about the need of application of bias correction to climate models outputs, I refer to^22,25,48–50^. While investigating the climate change, it was noted that there were marked differences between observed and model’s simulated meteorological data. To bridge the two data sets, a statistical bias correction technique is implemented. Different statistical methods are available for bias correction, however, in this study I will exploit the Quantile Delta Mapping (QDM) method as it preserves the relative and absolute climate change in the future projections^42^. N-dimensional QDM for statistical bias correction^51^ has used which preserve the spatial dependence structure between climate variables by implementing it to climate data at multiple sites simultaneously. This method comprises four steps to accomplish bias correction. The initial step is the calculation of cumulative distribution function (CDF) of the model projected data ${\mathrm{Y}}_{\mathrm{m},\mathrm{p}}$. In this study it is assumed that $\mathrm{h},\mathrm{ o},\mathrm{ m},\mathrm{ p}$ stand for historical, observed, model’s baseline, model’s projected data, respectively. Further, $\mathrm{Y}$ and $\mathrm{\varphi }$ represent the data and CDF of the data, respectively. The CDF of model predicted data is given in Eq. (1).

1 $${\mathrm{\varphi }}_{\mathrm{m},\mathrm{p}}\left(\mathrm{y}(\mathrm{t})\right)=\mathrm{P}\left({\mathrm{Y}}_{\mathrm{m},\mathrm{p}}\left(\mathrm{t}\right)\le \mathrm{y}(\mathrm{t})\right)$$

Find the relative change using the ratio of the quantile function (inverse CDF) of model predicted data implemented to the CDF of model predicted data and the quantile function of baseline simulated data applied to model predicted data as represented by Eq. (2)

2 $${\Delta }_{\mathrm{m}}\left(\mathrm{y}(\mathrm{t})\right)=\frac{{{\mathrm{\varphi }}_{\mathrm{m},\mathrm{p}}}^{-1}\left({\mathrm{\varphi }}_{\mathrm{m},\mathrm{p}} (\mathrm{y}\left(\mathrm{t}\right))\right)}{{{\mathrm{\varphi }}_{\mathrm{m},\mathrm{h}}}^{-1}\left({\mathrm{\varphi }}_{\mathrm{m},\mathrm{p}}(\mathrm{y}\left(\mathrm{t}\right))\right)}= \frac{{\mathrm{y}}_{\mathrm{m},\mathrm{p}}\left(\mathrm{t}\right)}{{{\mathrm{\varphi }}_{\mathrm{m},\mathrm{h}}}^{-1}\left({\mathrm{\varphi }}_{\mathrm{m},\mathrm{p}}(\mathrm{y}\left(\mathrm{t}\right))\right)}$$

The quantiles of model’s predicted data ${\mathrm{\varphi }}_{\mathrm{m},\mathrm{p}}(\mathrm{y}\left(\mathrm{t}\right))$ can now be corrected by implementing the inverse CDF estimated from observational data as expressed in Eq. (3).

3 $${\widehat{\mathrm{Y}}}_{\mathrm{o},\mathrm{m}}\left(\mathrm{t}\right)={{\mathrm{\varphi }}_{\mathrm{o},\mathrm{h}}}^{-1}\left({\mathrm{\varphi }}_{\mathrm{m},\mathrm{p}}(\mathrm{y}\left(\mathrm{t}\right))\right)$$

The final bias corrected data of future projections can be obtained by implementing the relative changes to the historical bias corrected data as given in Eq. (4).

4 $${\widehat{\mathrm{Y}}}_{\mathrm{m},\mathrm{f}}\left(\mathrm{t}\right)={\widehat{\mathrm{Y}}}_{\mathrm{o},\mathrm{m}}\left(\mathrm{t}\right) . {\Delta }_{\mathrm{m}}(\mathrm{y}\left(\mathrm{t}\right))$$

${\widehat{\mathrm{Y}}}_{\mathrm{m},\mathrm{f}}\left(\mathrm{t}\right)$ is the future model’s bias corrected data, however, to preserve absolute changes (for instance, temperature), Eqs. (2) and (4) can be applied additively rather than multiplicatively^42^. For further details about QDM, we refer to^42,52^.

### Watershed model

**Figure 3:**
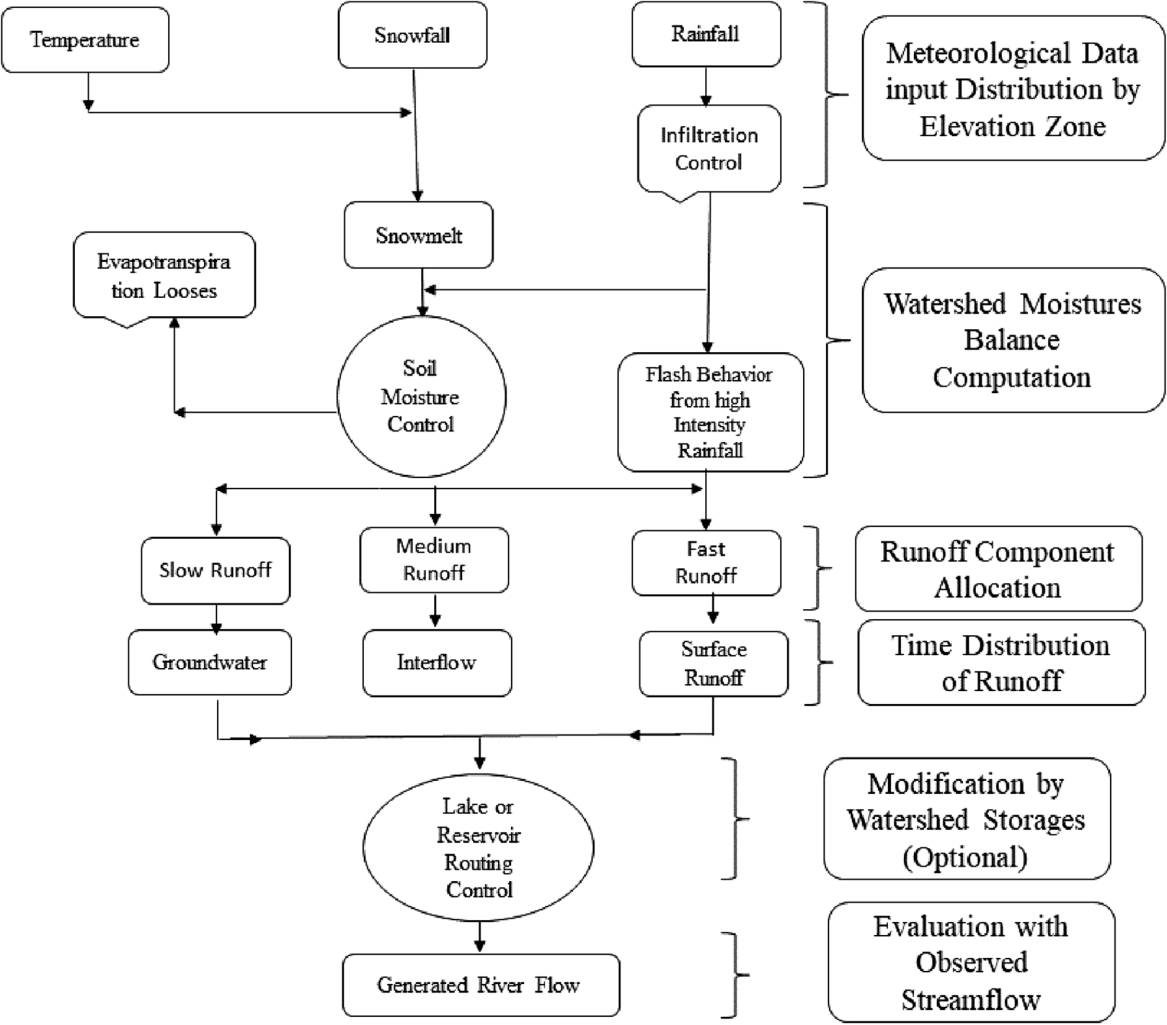
Schematic detail of the University British Columbia Watershed Model (UBCWM).

The Watershed Model developed by Quick and Pipps^37^ at the University of British Columbia and known as University of British Columbia Watershed Model (UBCWM) has been employed in this study. The model has been further improved with the time by including various user-friendly features^39^. The UBCWM is semi-distributed energy-balance model with the capability to simulate pluvial, nival and glacial runoff on hourly and daily basis with minimum hydrometeorological data requirement. The UBCWM conceptualizes the target watershed as the number of elevation bands or zones. Each elevation zone has variable characteristics and land use, for instance, forest, land use, impermeable, open, glaciated areas. The model allows the users for station-wise correction factors for vertical and horizontal precipitation’s distribution while temperature (minimum and maximum) is distributed by using adequate lapse rate across the basin^53^. The model determines whether the precipitation is snow or rain, based on temperature and calculate the snowpack accumulation as a function of elevation^54^. The UBCWM has a comprehensive snowmelt routine, for which the required parameters are pre-calibrated for the HKH environment^55^. A simplified energy budget approach is used in UBCWM that use limited data on minimum and maximum temperature to estimates the snowmelt^39^. The generated runoff from different components (rainfall, snowmelt and glacier melt) is distributed into four runoff components and achieved by the soil moisture mechanism^53,54^. These four runoff components, the fast (surface runoff), the medium (interflow runoff), the slow (upper zone groundwater runoff) and the very slow (deep zone groundwater runoff) are simulated by using the linear storage technique. By using this approach, the model calculates the runoff form each land use portion of each band and it is added to the generated runoff from other land use portions of the band to estimate the runoff for the elevation band. This procedure is repeated for each elevation band and the sum of the runoff from all elevation band is the runoff of the watershed for time step (hourly or daily). The UBCWM has more than 90 parameters, however, application of the model to various climatic regions and experience shown that a limited number of parameters need to be optimized and majority of the parameters take the standard default (constant) values^54,55^. A list of the parameters optimized in this study is given in the supplementary material labeled as S3. The UBCWM has been widely used for river flow projection, particularly in mountainous watersheds in different parts of the world^37,56–59^. UBCWM was chosen among the top-ranked medium complexity hydrologic model for mountainous catchments in different model-intercomparison studies^56,60^. More detail about UBCWM is presented schematically in Fig. 3 which show six different components of the model.

### Forcing UBNWM with meteorological data

The UBCWM hydrologic model is used for river flow projections. The meteorological observations over the UIB, upstream of Tarbela Reservoir for forcing UBCWM while river flow data from Besham Qilla gauging station, located upstream of Tarbela Reservoir, have been used for calibration and validation of the UBCWM. The model is calibrated for the period of 1990–1999 and validated for the duration of 2000–2004. The model’s performance is measured by using coefficient of determination (${\mathrm{R}}^{2}$) and Nash–Sutcliffe efficiency statistics. For details about these efficiency statistics, we refer to^11,12,61–66^ and for further details about the evaluation of watershed model to^67^. For hydrological projections, the outputs of regional climate model (Conformal-Cubic Atmospheric Model = CCAM) at 50 × 50 km horizontal resolution over the target location have been used. For the assessment of the potential changes in the future, the CCAM simulated data is divided into four durations. Baseline: 1976–2005; Future-01: 2011–2040; Future-02: 2041–2070; Future-03: 3071–2100. A comparison has been made between each future’s period with the baseline duration.

### Forecasting outflow by using BDLM

There are different classes of time series models which can be used to identify a data generating process and then for forecasting. These models including the popular class of Autoregressive Integrating Moving Average (ARIMA) models^68,69^, a non-linear class of Autoregressive Conditional Heteroscedasticity (ARCH) and Generalized ARCH (GARCH) models^70,71^ which have a multitude of applications in the real life^30,72,73^. However, besides other requirement these models required the stationarity of the time series data. If the data is non-stationary, then we need some sort of transformation to make it stationary. There are some time series models which don not need such type of requirement. Bayesian Dynamic Linear Model (BDLM) is a time series model which can be applied to non-stationary data, data with structural breaks or data with sudden jumps etc. Let assume that the outflow from the reservoir is denoted by ${O}_{t}$, then the BDLM can be expressed by Eqs. (5, 6). It is assumed that unobserved states are: ${\theta }_{1}, {\theta }_{1}, \dots , {\theta }_{t}$ observed river flow: ${O}_{1}, {O}_{2}, \dots , {O}_{t}$

5 $${O}_{t}= {F}_{t}{\theta }_{t}+ {\vartheta }_{t}\quad {\vartheta }_{t}\sim N(0, {V}_{t})$$

6 $${\theta }_{t}= {G}_{t} {\theta }_{t-1}+ {\varphi }_{t} \quad {\varphi }_{t}\sim N(0, {W}_{t})$$

The ${\vartheta }_{t}$ and ${\varphi }_{t}$ are two independent white noise error terms for observation equation (Eq. 5) and state or system equation (Eq. 6), respectively. These error terms ${\vartheta }_{t}$ and ${\varphi }_{t}$ are independent both within each other and between them with known covariances ${V}_{t}$ and ${W}_{t}$, respectively, and zero mean. In addition, assumed that ${\theta }_{0}$ follow Gaussian distribution, i.e.,

$${\theta }_{0}\sim N({m}_{0}, {C}_{0})$$

In Eqs. (5, 6), ${{\varvec{\theta}}}_{{\varvec{t}}}$ represents a vector of unobserved states of the system of length k that are supposed to evolve with time according to state transition ${{\varvec{G}}}_{{\varvec{t}}}$ (the linear system operator), a matrix of order $k\times k$. For a time series data, the states (different features) can be seasonality or regressive, trend components^74,75^. In Eq. (5), we observe a linear combination of the states with a matrix ${{\varvec{F}}}_{{\varvec{t}}} (k\times p)$ which works as observation operator and transforms the model states to a time series observation. Based on observed river flow, the future’s forecast has been made by using BDLM. For further details about BDLM, I refer to^32,76,77^.

### Assessment of water availability and response of the reservoir

Water availability is investigated by comparing the future available water with the baseline period on monthly/daily basis graphically as well as numerically. Additionally, % changes in river flow have also been assessed on monthly basis for both considered climate change scenarios. For numerical assessment of water availability, Tarbela Dam is used as a measurement tool. Different parameters of the dam including dead level storage, maximum operating storage, discharge capacity of tunnels, discharge capacity of spillways and evapotranspiration are utilized to accomplish this task. The following balancing equation given in Eq. (7) has been used for this purpose:

7 $$\begin{gathered} {\text{Str}}_{{{\text{t}} + 1}} = {\text{Str}}_{{\text{t}}} + {\text{Inf}}_{{\text{t}}} - {\text{Otf}}_{{\text{t}}} - {\text{Evt}}_{{\text{t}}} \hfill \\ {\text{Otf}}_{{{\text{ext}}}} = \left\{ {\begin{array}{*{20}c} {\begin{array}{*{20}c} { = 0} \\ { > 0} \\ \end{array} \;\begin{array}{*{20}c} {if\quad {\text{Str}}_{{{\text{t}} + 1}} \le 6MAF} \\ {if\quad {\text{Str}}_{{{\text{t}} + 1}} > 6MAF} \\ \end{array} } \\ \end{array} } \right. \hfill \\ \end{gathered}$$

where ${\mathrm{Str}}_{\mathrm{t}+1}$ is the storage of the reservoir at time $\mathrm{t}+1$ (future), ${\mathrm{Str}}_{\mathrm{t}}$ is the storage of the reservoir at $\mathrm{t}$ (at present), ${\mathrm{Inf}}_{\mathrm{t}}$ is inflow (river flow) at time $\mathrm{t}$, ${\mathrm{Otf}}_{\mathrm{t}}$ is outflow from the reservoir at time $\mathrm{t}$ and ${\mathrm{Evt}}_{\mathrm{t}}$ is the evapotranspiration from the reservoir at time $\mathrm{t}$. MAF stands for million acre feet in second part of Eq. (7). The second part of the equation determine the extra outflow (other than the outflow used in part one of this equation) which will be further used to investigate that how much time we will need to open the spillways of the reservoir. In Eq. (7), ${\mathrm{Inf}}_{\mathrm{t}}$ is projected by using UBCWM, ${\mathrm{Otf}}_{\mathrm{t}}$ is forecasted by using BDLM and ${\mathrm{Evt}}_{\mathrm{t}}$ is calculated by using the methods of^31^. The extra outflow $\left({\mathrm{Otf}}_{\mathrm{ext}}\right)$ in part 2 of Eq. (7) can be used together with the outflow in part 1 of Eq. (7) to investigate the water availability and operation of the reservoir under the considered climate change scenarios.

## Results

The findings of this study are divided into different subparts to make it easy for understanding and presented in the subsequent subsections:

### Climate change

The average results about climate change for different seasons under both scenarios are presented in Table 2. We can see that the maximum temperature increases except for JJA and SON where there is no change during the 2011–2040 period under RCP4.5. It is noted that maximum increase is 1.5 °C (in MAM) and 2 °C (in MAM) during the 2041–2070 and 2071–2100 periods, respectively, under RCP4.5. Under RCP8.5, maximum increase is 1.1 °C (in MAM and JJA), 3.1 °C (MAM) and 6.4 °C (JJA) during the 2011–2040, 2041–2070 and 2071–2100 periods, respectively. There is higher increase in minimum temperature as compared to maximum temperature in the twenty-first century. Maximum increase in minimum temperature is 1.3 °C (in MAM), 1.9 °C (in MAM and DJF) and 2.6 °C (in MAM) during the 2011–2040, 2041–2070 and 2071–2100 periods, respectively, under RCP4.5. Meanwhile maximum changes are 3.9 °C (in MAM), 5.7 °C (in MAM) and 6.7 °C (in DJF) during the 2011–2040, 2041–2070 and 2071–2100, respectively, under RCP8.5. There is 5.1% (in DJF) decrease in precipitation during 2011–2040 with respect to the baseline period, however, maximum increase is 26.6% and 30.1% during the 2041–2070 and 2071–2100 periods, respectively, in SON under RCP4.5. Under RCP8.5, maximum increase is 16.1% (in SON), 24.5% (in SON) and 33.2% (in SON) during the 2011–2040, 2041–2070 and 2071–2100, respectively, however, a decrease of 1.2% is also observed during the 2041–2070 in DJF.

**Table 2:** Changes in maximum, minimum temperature and precipitation with respect to the baseline period (1976–2005) for different seasons under the RCP4.5 and RCP8.5.

Duration/seasons	DJF	MAM	JJA	SON	DJF	MAM	JJA	SON
	**Changes in Max-temperature compared with 1976–2005 for RCP4.5**				**Changes in Max-temperature compared with 1976–2005 for RCP8.5**			
2011–2040	0.2	0.6	0.0	0.0	0.8	1.1	1.1	0.7
2041–2070	1.4	1.5	1.1	0.9	2.9	3.1	3.2	2.7
2071–2100	1.8	2.0	1.8	1.3	5.8	6.3	6.4	5.0
	**Changes in Min-temperature compared with 1976–2005 for RCP4.5**				**Changes in Min-temperature compared with 1976–2005 for RCP8.5**			
2011–2040	0.3	1.3	0.9	0.7	3.5	3.9	3.8	3.0
2041–2070	1.9	1.9	1.6	1.7	5.6	5.7	5.6	4.9
2071–2100	2.4	2.6	2.2	2.2	6.7	5.9	5.4	5.1
	**Percent changes in precipitation compared with 1976–2005 for RCP4.5**				**Percent changes in precipitation compared with 1976–2005 for RCP8.5**			
2011–2040	− 5.1	8.4	14.9	21.9	7.6	11.6	8.9	16.1
2041–2070	11.5	5.7	9.4	26.6	− 1.2	14.2	16.1	24.5
2071–2100	3.2	8.8	8.5	30.1	3.5	32.9	13.0	33.2

The unit of change in temperature is ^0^C while for precipitation percent changes are presented.

The changes are in comparison to the baseline duration (1976–2005).

### Calibration and validation of watershed model

The UBCWM is calibrated and validated for the period of 1990–1999 and 2000–2004, respectively, using daily hydrological and meteorological observational data. The calibration process is completed after a number of experiments where different parameters of the model were tuned. The calibration process was stopped when no significant improvement was observed in consecutive experiments and concluded that the model is calibrated well. The efficiency statistics were higher during the validation period $\left({\mathrm{R}}^{2}=89{\%},\mathrm{Nash}-\mathrm{Sutcliffe}=90{\%}\right)$ as compared to the calibration period $\left({\mathrm{R}}^{2}=87{\%},\mathrm{Nash}-\mathrm{Sutcliffe}=89{\%}\right)$. The details about the efficiency measures of the calibration and validation of UBCWM is mentioned in supplementary material labeled as S2.

### Water availability and response of Tarbela Reservoir

**Figure 4:**
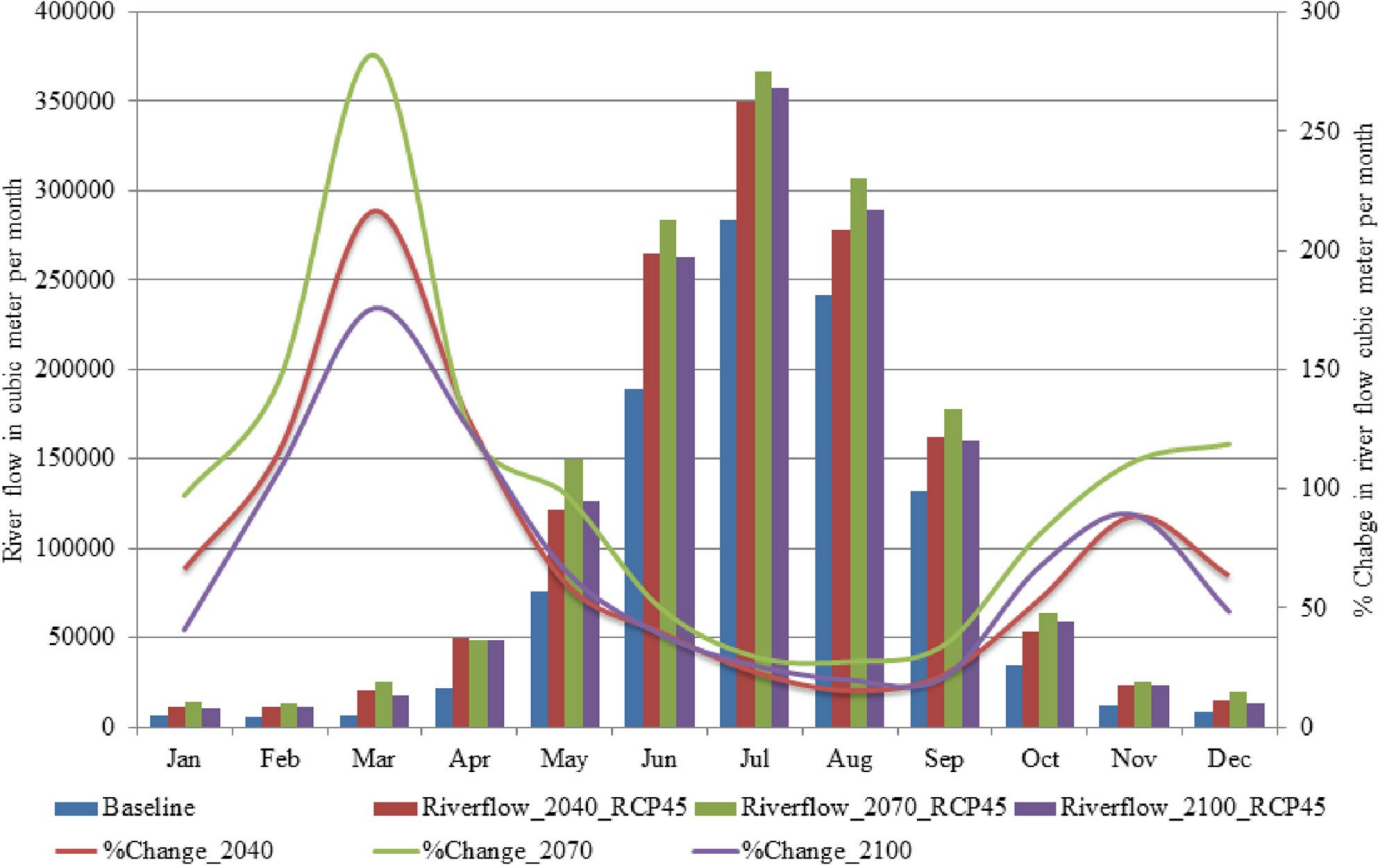
Projected river flow for the baseline (1976–2005) and future durations (2011–2040, (2041–2070, 2071–2100), change in river flow %Change_2040 (2011–2040), %Change_2070 (2041–2070) and %Change_2100 (2071–2100) in comparison to the baseline duration under the RCP4.5. Riverflow_2040, Riverflow_2070 and Riverflow_2100 represent river flow for the durations of 2011–2040, 2041–2070 and 2071–2100, respectively. The unit for river flow and change in the river flow is cubic meter per month.

Figure 4 presents information about monthly water availability and percent changes in river flows in different future time periods (2011–2040, 2041–2070, 2071–2100) with respect to the baseline period (1976–2005) under RCP4.5. It can be seen that the river flow is increasing during each future time period, however, interestingly in the last future time period it is decreasing in every month. Maximum increase has been observed during the second future time period for all months except April where the increase during the second future time and last future periods is almost the same. One possible reason for this shift in changes may be the contribution of glacier melt. Due to the rise in temperature, glacier melt can contribute maximum during the first and second future time periods and may be due to the shrinking glacier’s size, during the last future time period, the contribution may be reduced as compared to the earlier future time periods.

**Figure 5:**
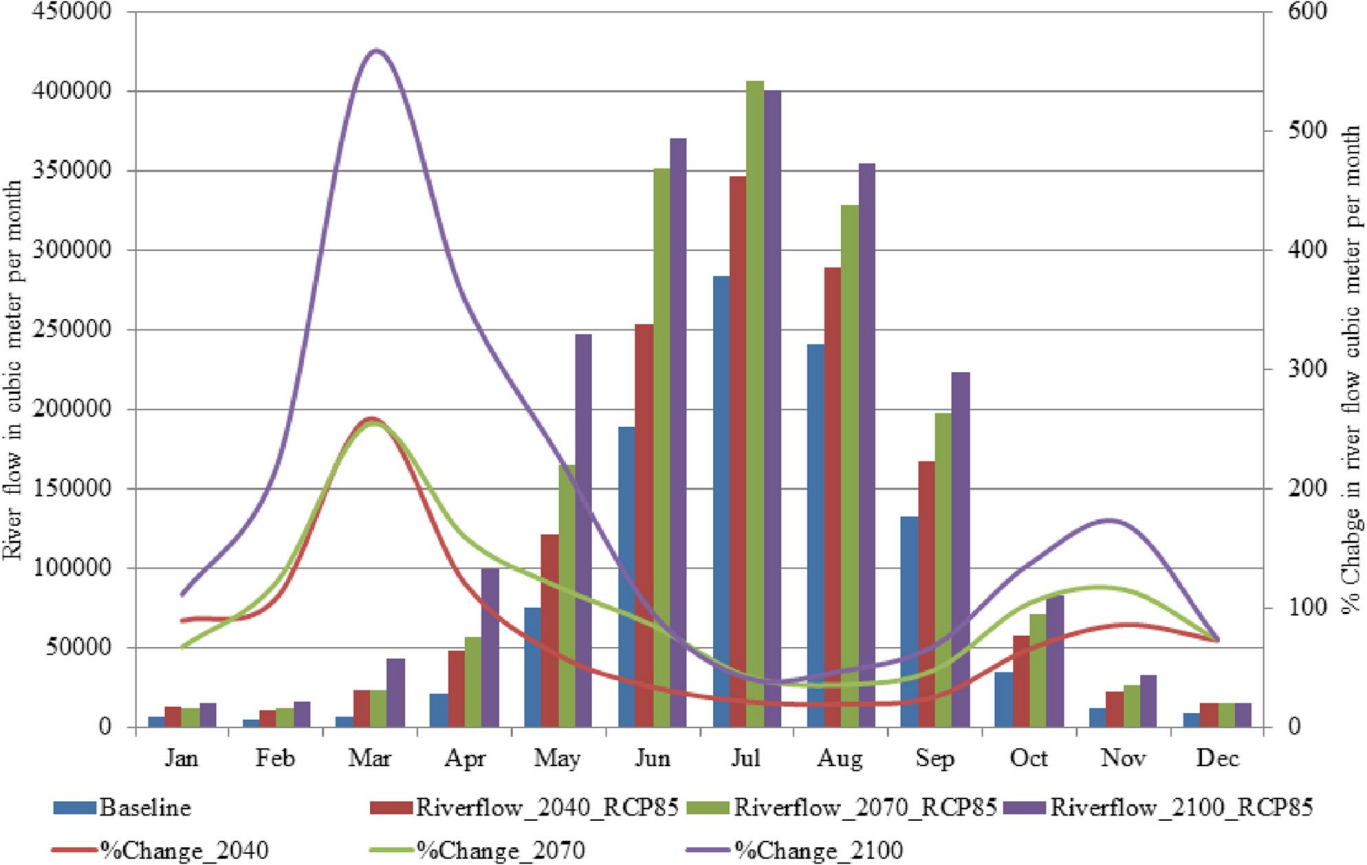
Similar to Fig. 4 but using the RCP8.5 scenario.

Figure 5 provides detailed information on water availability and their changes during future time periods as compared to the baseline period under RCP8.5. It can be seen that RCP8.5 behaves differently from RCP4.5 in terms of water availability. It can be noted that maximum increase (in percent) is observed during the last future time period while it was observed in the second future time period under RCP4.5. Maximum increase is noted during 2071–2100 in all months except July and December where in July maximum increase is observed during the 2041–2070 while in December, the maximum increase is almost the same during all considered three future periods. It is also noted that in both climate change scenarios, the maximum increase (in percent) is observed during winter season rather than during summer season. From Figs. 4 and 5, we can infer that more water will be available in the future as compared to the baseline period, however, the demand of water may be increased from various sectors due to increase in population, urbanization and industrialization etc. Therefore, proper management of the available water may be a challenge in future.

Table 3 presents important information about water availability and reservoir’s response to changing climate for both scenarios. The entries in third column are about how many times the dead level storage is hit by the water surface for each scenario. During the baseline period the dead level has been hit 58 times while during the future time periods the number of times changed to 19, 8 and 16 for first, second and third future period, respectively, under RCP45. The fourth column shows that how much time the maximum operating storage has been crossed. It is obvious that during the baseline period the maximum operating storage of the reservoir is crossed 102 times out of 360 times (360 months in 30 years). During the future time periods it is changed to 195, 244, 199 during the first, second and third future period, respectively, for RCP4.5. The entries in the fifth column represent that how many times the total discharge capacity of the tunnels has been crossed. It can be seen that it is crossed one time during the baseline time period, however, it is increased to 19, 23 and 17 times during the first, second and third future period, respectively, for the RCP4.5. This show that under the RCP4.5, there will be more water available during the first and second future period, however, there is decreasing trend in water availability during the third future time period as compared to the first two future periods. The numbers in the fifth column also indicate that probably we will need to open spillways (the mentioned number of times) to spill out the extra water to avoid overtopping of the reservoir.

**Table 3:** Important information about water availability and response of Tarbela Reservoir under changing climate in the Upper Indus Basin, Pakistan.

Scenarios	Duration/parameters	Dead Level Storage (1.94 MAF)	Maximum operating storage (6.00 MAF)	Total discharge by tunnels (26.19 MAF/month)	Total discharge by tunnels and spillways (26.19 + 89.26 = 115.45 MAF/month)
RCP4.5	1976–2005	58	102	01	0
	2011–2040	19	195	19	0
	2041–2070	8	244	23	0
	2071–2100	16	199	17	0
RCP8.5	1976–2005	58	102	01	0
	2011–2040	16	207	14	0
	2041–2070	7	250	53	0
	2071–2100	2	288	57	0

The entries in columns 3–6 show the number of times exceed the parameter of the reservoir mentioned on the top of each column under each climate change scenarios for different time periods.

For RCP8.5 the results are different than the results of RCP4.5 as there is consistent increasing trend in water availability during all three future periods. During the baseline period the dead level storage has been hit 58 times while during the future time periods the frequency changed to 16, 7 and 2 for first, second and third future period, respectively. From the fourth column under RCP8.5, it can be seen that during the baseline period the maximum operating storage of the reservoir is crossed 102 times out of 360 times. In the future time periods, it is changed to 207, 250, and 288 during the first, second and third future period, respectively. From the fifth column it can be seen that the total discharge capacity has been crossed by outflow of the reservoir one time during the baseline time period, however, it is increased to 14, 53 and 57 during the first, second and third future period, respectively. This also indicates that under the RCP8.5, there will be more water available during all considered future periods and behave differently as compared to the RCP4.5.

The last column of Table 3 shows that how much time the combined discharge capacity of tunnels and spillways has been crossed. Alternatively, we can say that how much time the reservoir has been overtopped. Luckily the findings show that there are no chances of overtopping of Tarbela Reservoir till the end of this century under the considered climate change scenarios.

Most of the statistics in Table 3 show an increasing trend in water availability which is good for Pakistan, however, mismanagement can worsen the situation as observed in the past^78^.

**Figure 6:**
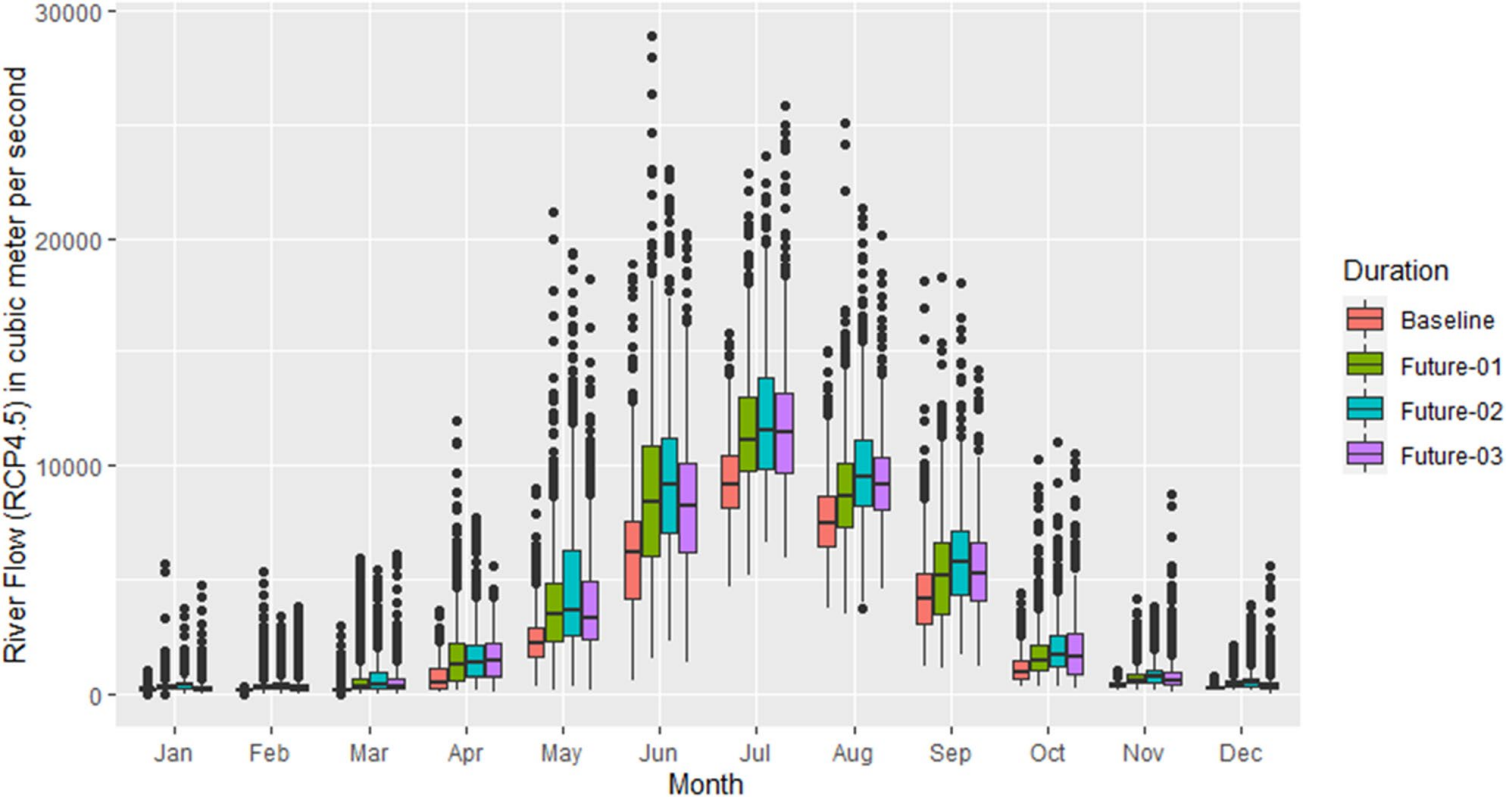
Monthly comparison of daily reiver flow in future with the baseline (1976–2005) under the RCP4.5. On x-axis, moths are mentioned while on y-axis, river flow is presented in cubic meter per second. The future durations are: Future-01 = 2011–2040, Future-02 = 2041–2070, Future-03 = 2071–2100.

**Figure 7:**
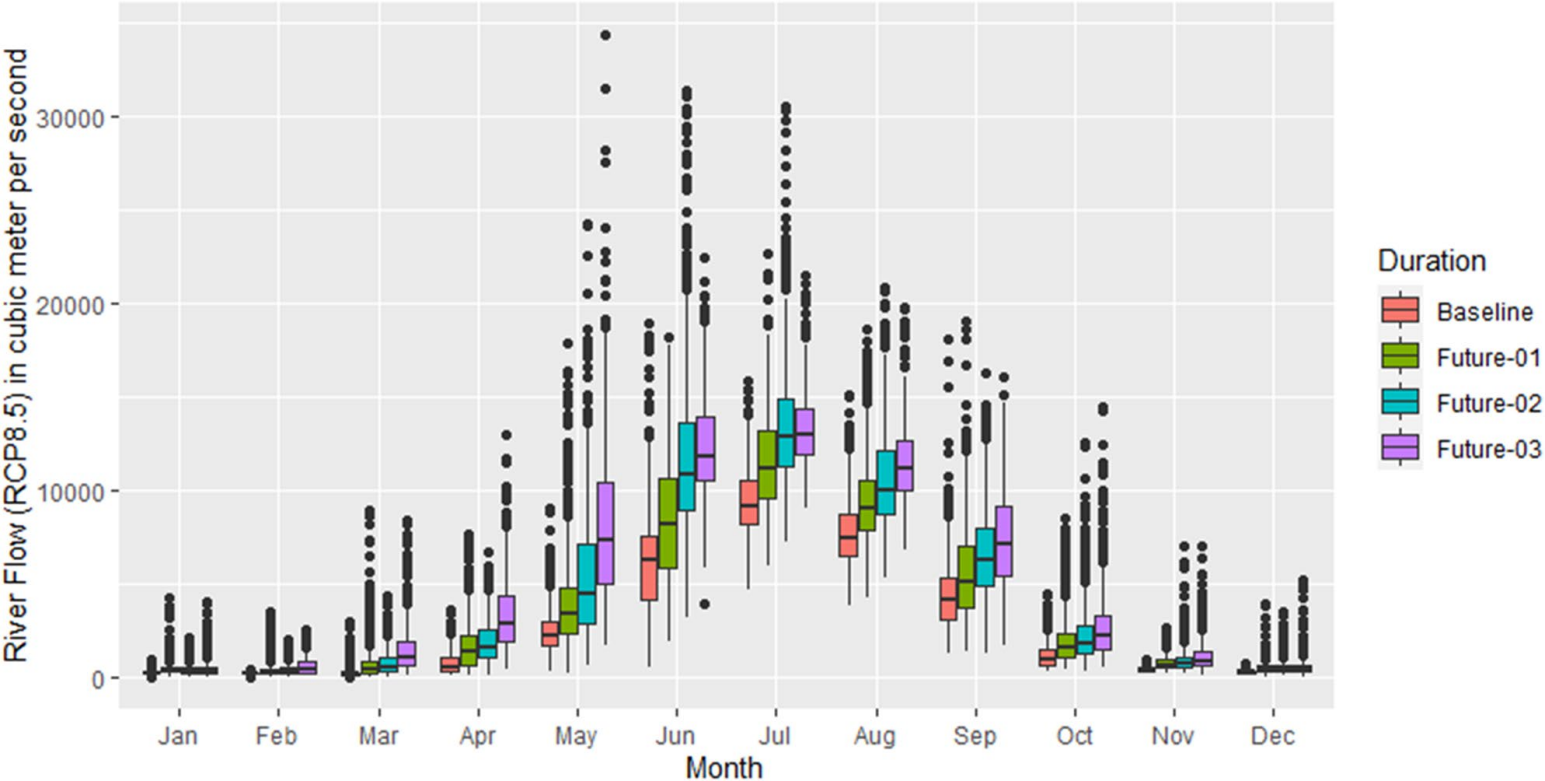
Similar to Fig. 6 but using the RCP8.5 scenario.

Figures 6 and 7 show the comparison between daily river flow for each month of the future period with the baseline for the RCP4.5 and RCP8.5, respectively. It can be noted from Fig. 6 that the average river flow has been consistently increases during the 2011–2040 and 2041–2070, however, during 2071–2100, the river flow decreases as compared to the previous time periods. This pattern is noted during all months of the year except the months of April and October. It can be observed that there are more outliers during the winter season as compared to the summer, however, the highest value is recorded during the 2011–2040 in the month of June. Similarly, the results in Fig. 7 represents a comparison of the futures’ river flow with the baseline for the RCP8.5. The average river flow increases consistently during the future periods with respect to the baseline, however, in the month of July during 2071–2100, the river flow decreases as compared to the previous future periods. The highest river flow is recorded in the month of May during 2071–2100 which indicates a shift towards left side of the distribution as compared to RCP4.5.

**Figure 8:**
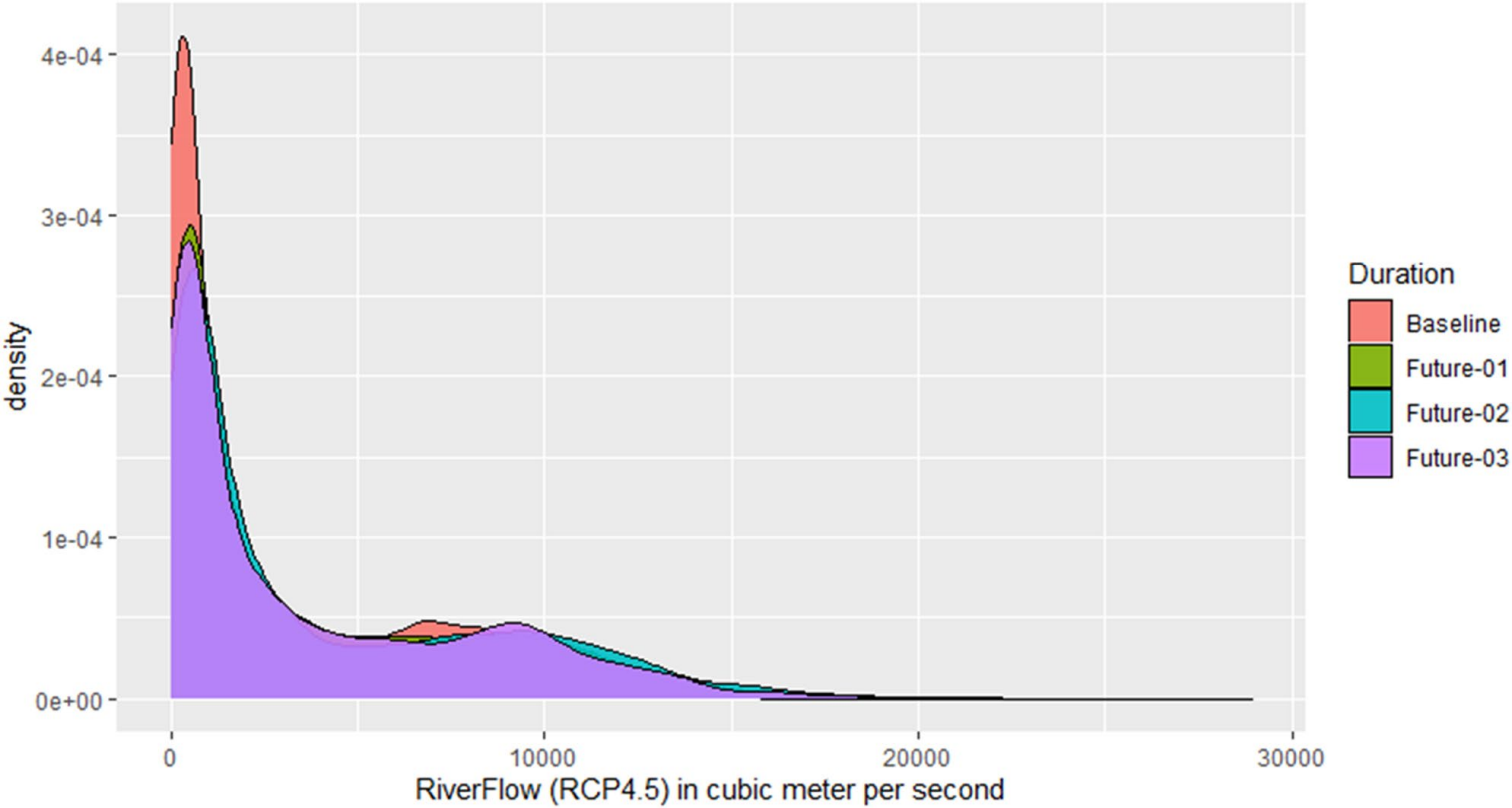
Comparison of probability distribution of the futures’ river flow with the baseline (1976–2005) for RCP4.5. River flow is mentioned on x-axis in cubic meter per second while their density is mentioned on y-axis. The future durations are: Future-01 = 2011–2040, Future-02 = 2041–2070, Future-03 = 2071–2100.

**Figure 9:**
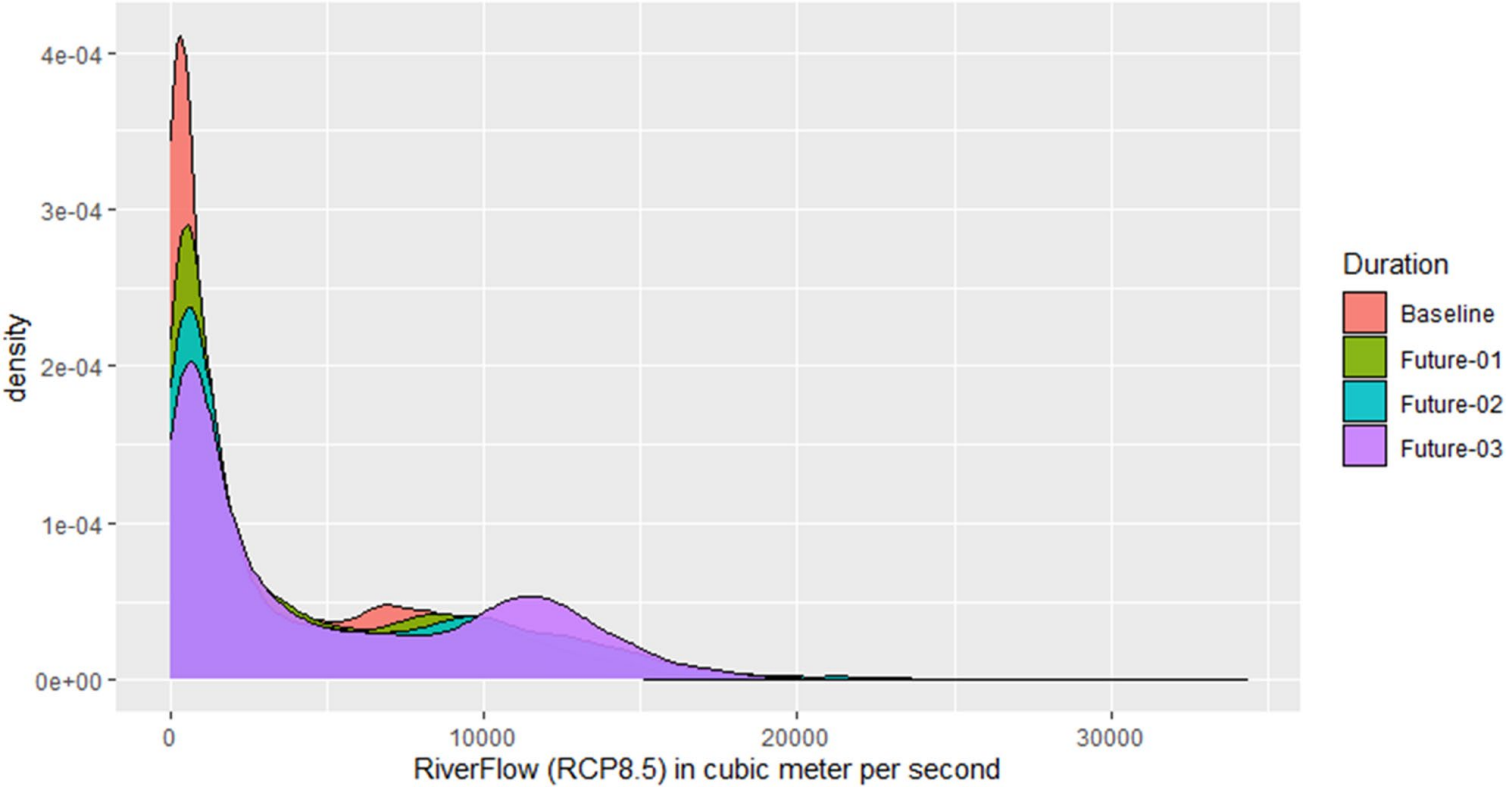
Similar to Fig. 8 but using the RCP8.5 scenario.

Figures 8 and 9 show a comparison of the future’s simulated daily river flows to the baseline river flow for RCP4.5 and RCP8.5, respectively, by using the probability density functions. There is a shift in the average river flow as well as in their variability under the RCP4.5. Under RCP8.5, the shift in the average river flow is clearer and more consistent toward right side as compared to RCP4.5. The highest values are dominated by the period of 2071–2100, however, there are some highest values during the 2041–2070 beyond 20,000 cubic meter per second.

## Discussion and conclusions

As UIB comprises one of the largest non-polar glaciers in the world and therefore, its hydrological regime is very sensitive to increases in summer temperature. For example, Archer^15^ noted that the river flow can results 16–17% increase in response to 1 °C increase in mean summer temperature. Different studies reported similar results, however, using different approaches regarding water availability. For instance, Khan et al.^11^ investigated water availability during the first half of twenty-first century and concluded that enough water will be available in this period. Ali et al.^12^ reported that there will be more water available in the future as compared to the baseline period. Khan et al.^13^ investigated water availability during the 2011–2040 and concluded that enough water will be available in this period under the RCP4.5 and RCP8.5. In a recent study, Kiani et al.^59^ developed hydrological projections under the Paris agreement (PA) warming targets over the UIB. They concluded that the PA warming targets are probably exceed a decade earlier in Pakistan as compared to the globe. In addition, they noted increase in the river flow under RCP4.5 and RCP8.5. Shukla et al.^79^ concluded in their study that in future, the river flow may increase by 11–19% under the RCP4.5 and RCP8.5, respectively, by utilizing the outputs of CCSM4 (a regional climate model). Shah et al.^80^ noted increase in the river flow in their study based on the output of an RCM with the RCP4.5 and RCP8.5 in the UIB. The IPCC also reported that the regions like South Asia and some parts of Northern Europe will most probably experience increases in water availability. However, other parts of the world which are dry comparatively, for instance, South Africa, the Mediterranean region and South America will probably face further decrease in water availability^81,82^. Climate extremes like heat wave and drought are projected over different parts of the country^20,83–85^. However, various studies suggested that the northern part of Pakistan is more vulnerable to the changing climate and reported a higher rise in temperature which may result a rise in glaciers and snow melt in the northern Pakistan^12,86^.

The demand of water may be increased in the future due to different potential phenomena. For instance, shift in patterns during the recent decades in large scale circulations affect particularly the arid and semi-arid region of the globe facing moderate to sever droughts which further affect the environmental and socio-economic conditions^87^. In addition, the substantial reliance of Pakistan rain-based agriculture intensifies the vulnerability of Pakistan to food scarcity due to droughts. Shahzaman et al.^88^ investigated about agriculture drought using different data sets and concluded that the spatio-temporal drought characterization is likewise over the South Asian region. Climate extremes like drought events, heavy precipitation have been increased in different parts of the world including Pakistan^89,90^. Therefore, it is important to manage the available water wisely to avoid water crises in the near future. Towards this end, my recommendations are given at the end of this section.

It is noted that maximum and minimum temperature are increases in the future under both climate change scenarios. Maximum increase in maximum temperature is noted in MAM (2 °C) and JJA (6.4 °C) under RCP4.5 and RCP8.5, respectively, during 2071–2100. Maximum increase in minimum temperature is noted in MAM (2.6 °C) and DJF (6.7 °C) under RCP4.5 and RCP8.5, respectively, during 2071–2100. Precipitation decreases 5.1% in DJF under RCP4.5 during 2011–2040 while it increases by 33.2% in SON under RCP8.5 during 2071–2100.

Results about water availability show that enough water (more than the available water during the baseline time period) is available, however, it is decreases in some months and in different future periods. In RCP4.5, there is increasing trend in water availability during first and second future time periods while during the third (last) future time period, there is decreasing trend in all months as compared to the previous future periods. Unlike the RCP4.5, under RCP8.5, there is consisting increasing trend in water availability in all future time periods as compared to the baseline. The increasing pattern is almost the same under both RCPs as there is maximum increase during the winter season and minimum increase during the summer season.

It is noted that enough water is available in a sense that the outflow will cross the capacity of tunnels of Tarbela Reservoir which is 26 MAF in a month by various number of times. Under both climate change scenarios, the number of times to hit dead level storage of the reservoir is decreasing; the number of times to cross the discharge capacity of the tunnels is increasing. Therefore, it will be probably necessary to open spillways to spill out the extra water after keeping the maximum operating storage of the reservoir. This shows that there will be more water available in the future periods as compared to the baseline period under both climate change scenarios. The outflow did not exceed the combined discharge capacity of tunnels and spillways under either scenario, therefore, there is no risk of overtopping of Reservoir till the end of twenty-first century for the considered scenarios. The following adaptation and mitigation strategies are suggested against climate change and optimal utilization of available water:

- Curriculum at school level should include material about climate change, impacts of climate change and importance of water so that the new generation have the awareness and can play their role about adaptation and mitigation strategies against the changing climate.
- Afforestation can play significant role in climate change mitigation. Therefore, it is suggested that the government should provide energy (gas and electricity) to maximum inhabitants and with nominal prices. This can help to stop deforestation in the country.
- Regarding water situation in the country, it is suggested that small, medium and large dams may be constructed for the storage of available water during high river flow (particularly in summer) season. The stored water can then be used during low river flow for agriculture and other purposes. Beside water storage, this can help to increase the hydropower generation and rise the groundwater’s surface in the country.

## Supplementary Information

Supplementary Information 1.

Supplementary Information 2.

## Data Availability

The datasets used and/or analyzed during the current study available from the corresponding author on reasonable request.
