# Supplementary material for: Water availability and response of Tarbela Reservoir under the changing climate in the Upper Indus Basin, Pakistan
Source: Sci Rep. 2022 Sep 23;12:15865. doi: 10.1038/s41598-022-20159-x (PMC9508107; doi:10.1038/s41598-022-20159-x)
Supplement: Supplementary file 1 — Supplementary Information 1. [file 41598_2022_20159_MOESM1_ESM.docx]

**List of Acronyms**

| S. No | Short Name | Full Name |
| --- | --- | --- |
| 1 | BDLM | Bayesian Dynamic Linear Model |
| 2 | CCAM | Conformal-Cubic Atmospheric Model |
| 3 | CDF | Cumulative Distribution Function |
| 4 | CMIP6 | Coupled Model Intercomparison Project Phase6 |
| 5 | CMIP6 | Coupled Model Intercomparison Project Phase6 |
| 6 | DEM | Digital Elevation Model |
| 7 | DJF | December January February |
| 8 | FAO | Food and Agriculture Organization |
| 9 | GCM | General Circulation Model |
| 10 | HKH | Himalaya Karakoram Hindukush |
| 11 | IMF | International Monitory Fund |
| 12 | IPCC | Intergovernmental Panel on Climate Change |
| 13 | IRSA | Indus River System Authority |
| 14 | JJA | June July August |
| 15 | MAF | Million Acre Feet |
| 16 | MAM | March April May |
| 17 | MW | Megawatt |
| 18 | PA | Paris Agreement |
| 19 | PMD | Pakistan Meteorological Department |
| 20 | QDM | Quantile Delta Mapping |
| 21 | RCM | Regional Climate Model |
| 22 | RCP | Representative Concentration Pathways |
| 23 | SDGs | Sustainable Development Goals |
| 24 | SON | September October November |
| 25 | UBCWM | University of British Columbia Watershed Model |
| 26 | UIB | Upper Indus Basin |
| 27 | UNDP | United Nation Development Program |
| 28 | USGS | United States Geological Survey |
| 29 | WAPDA | Water and Power Development Authority |
