# Supplementary material for: Water availability and response of Tarbela Reservoir under the changing climate in the Upper Indus Basin, Pakistan
Source: Sci Rep. 2022 Sep 23;12:15865. doi: 10.1038/s41598-022-20159-x (PMC9508107; doi:10.1038/s41598-022-20159-x)
Supplement: Supplementary file 2 — Supplementary Information 2. [file 41598_2022_20159_MOESM2_ESM.docx]

Supplementary Material

| 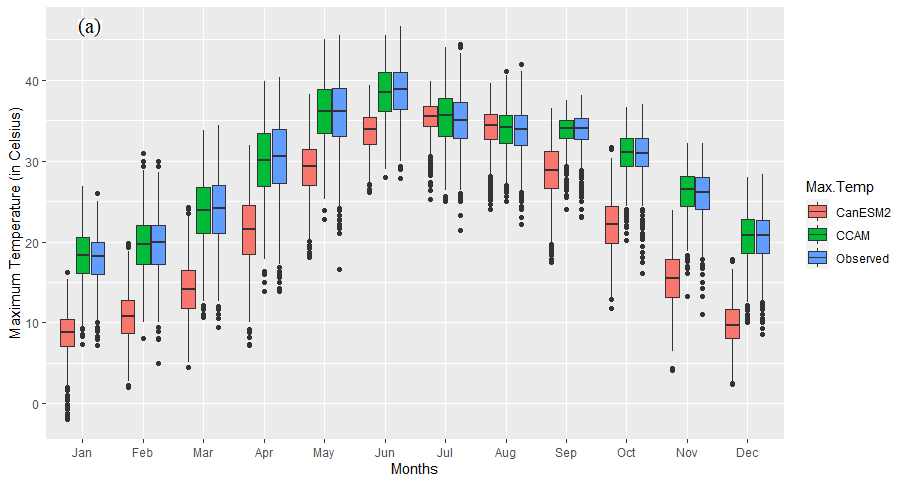 |
| --- |
| 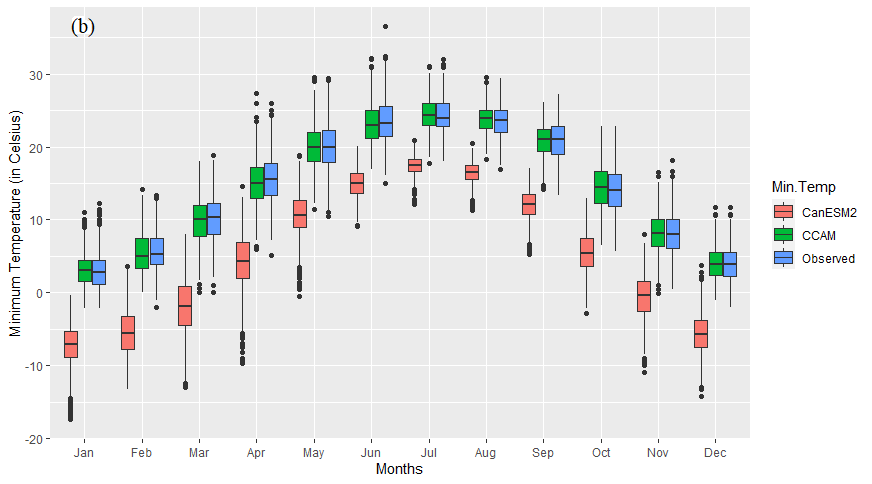 |
| 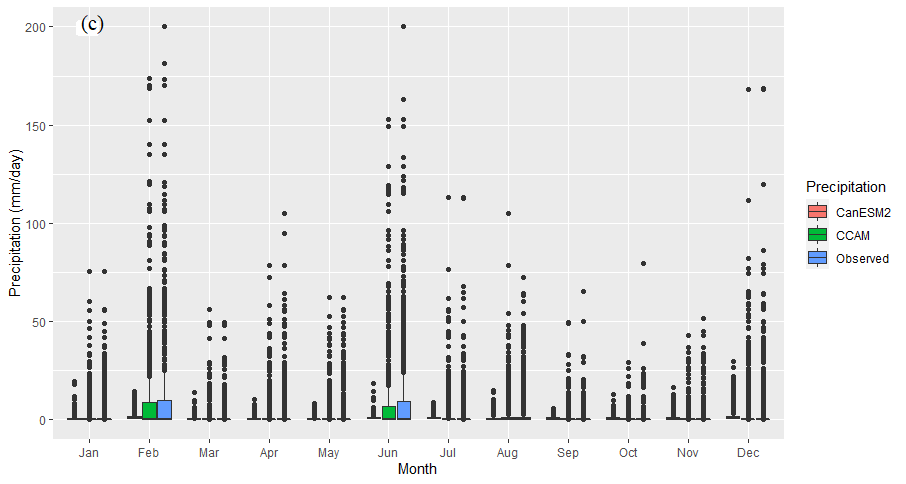 |

S1: This figure represents a comparison between the outputs of GCM (CanESM2) and RCM (CCAM) with observed data for the time period of 1976-2005. (a) maximum temperature, (b) minimum temperature (c) precipitation.

| S. No | Year | Coeff. of Eff. | Coeff. of Det. | |
| --- | --- | --- | --- | --- |
| 1 | Oct-1995-Sep-1996 | 0.69 | | 0.78 |
| 2 | Oct-1996-Sep-1997 | 0.79 | | 0.79 |
| 3 | Oct-1997-Sep-1998 | 0.79 | | 0.84 |
| 4 | Oct-1998-Sep-1999 | 0.69 | | 0.83 |
| 5 | Oct-1999-Sep-2000 | 0.76 | | 0.76 |
| 6 | Oct-2000-Sep-2001 | 0.80 | | 0.80 |
| 7 | Oct-2001-Sep-2002 | 0.81 | | 0.82 |
| 8 | Oct-2002-Sep-2003 | 0.79 | | 0.88 |
| 9 | Oct-2003-Sep-2004 | 0.85 | | 0.85 |
|  | **Efficiency Statistics for the Calibration and Validation Periods** | | | |
| Calibration | Oct-1990-Sep-1999 | 0.87 | | 0.89 |
| Validation | Oct-2000-Sep-2004 | 0.89 | | 0.90 |

S2. This table presents the detail about the year-wise efficacy of the UBCWM for the calibration period (1995-2004). Where, Coeff. of Eff. Stands for coefficient of efficiency and Coeff. of Det. Stands for coefficient of determination.

S3. The list of parameters of UBCWM calibrated during calibration for this study.

| S. No | Parameter | Description | Range | Default Value | Value sed in this study |
| --- | --- | --- | --- | --- | --- |
| 1 | P0GRADL | Precipitation gradient factor for elevation below E0LMID, %. | 0 - 20 | 5 | 4 |
| 2 | P0GRADM | Precipitation gradient factor for elevation below E0LHI, %. | 0 - 20 | 0 | 0 |
| 3 | P0GRADL | Precipitation gradient factor for elevation above E0LHI, %. | 0 - 20 | 0 | 9 |
| 4 | P0SREP | Adjustment to precipitation when average temperature <0 (snowfall) | -1.0 – 1.0 | 0 | -0.941 – 0.2 |
| 5 | P0RREP | Adjustment to precipitation when average temperature > A0FORM (rain) | -1.0 – 1.0 | 0 | -0.8 – 0.0 |
| 6 | C0IMPA | Fraction of impermeable area in the band | 0 – 1.0 | 0.2 | 0.5 - 0.765 |
| 7 | P0PERC | Groundwater percolation in mm/day | 0-50 | 15 | 25 |
| 8 | P0DZSH | Deep zone share fraction | 0-1.0 | 0.5 | 0.5 |
| 9 | P0UGTK | Time constant for upper groundwater runoff (days) | 10-50 | 30 | 30 |
| 10 | P0DZTK | Time constant for deep groundwater runoff (days) | 100-300 | 150 | 150 |
